# Distinctive Association of the Functional Connectivity of the Posterior Cingulate Cortex on Memory Performances in Early and Late Amnestic Mild Cognitive Impairment Patients

**DOI:** 10.3389/fnagi.2021.696735

**Published:** 2021-07-01

**Authors:** Dong Woo Kang, Sheng-Min Wang, Yoo Hyun Um, Hae-Ran Na, Nak-Young Kim, Chang Uk Lee, Hyun Kook Lim

**Affiliations:** ^1^Department of Psychiatry, Seoul St. Mary’s Hospital, College of Medicine, The Catholic University of Korea, Seoul, South Korea; ^2^Department of Psychiatry, Yeouido St. Mary’s Hospital, College of Medicine, The Catholic University of Korea, Seoul, South Korea; ^3^Department of Psychiatry, St. Vincent’s Hospital, College of Medicine, The Catholic University of Korea, Suwon, South Korea; ^4^Department of Psychiatry, Keyo Hospital, Uiwang, South Korea

**Keywords:** functional connectivity, early mild cognitive impairment, late mild cognitive impairment, posterior cingulate cortex, memory performance

## Abstract

**Background:**

Attempts have been made to explore the biological basis of neurodegeneration in the amnestic mild cognitive impairment (MCI) stage, subdivided by memory performance. However, few studies have evaluated the differential impact of functional connectivity (FC) on memory performances in early- and late-MCI patients.

**Objective:**

This study aims to explore the difference in FC of the posterior cingulate cortex (PCC) among healthy controls (HC) (*n* = 37), early-MCI patients (*n* = 30), and late-MCI patients (*n* = 35) and to evaluate a group-memory performance interaction against the FC of PCC.

**Methods:**

The subjects underwent resting-state functional MRI scanning and a battery of neuropsychological tests.

**Results:**

A significant difference among the three groups was found in FC between the PCC (seed region) and bilateral crus cerebellum, right superior medial frontal gyrus, superior temporal gyrus, and left middle cingulate gyrus (Monte Carlo simulation-corrected *p* < 0.01; cluster *p* < 0.05). Additionally, the early-MCI patients displayed higher FC values than the HC and late-MCI patients in the right superior medial frontal gyrus, cerebellum crus 1, and left cerebellum crus 2 (Bonferroni-corrected *p* < 0.05). Furthermore, there was a significant group-memory performance interaction (HC *vs*. early MCI *vs*. late MCI) for the FC between PCC and bilateral crus cerebellum, right superior medial frontal gyrus, superior temporal gyrus, and left middle cingulate gyrus (Bonferroni-corrected *p* < 0.05).

**Conclusion:**

These findings contribute to the biological implications of early- and late-MCI stages, categorized by evaluating the impairment of memory performance. Additionally, comprehensively analyzing the structural differences in the subdivided amnestic MCI (aMCI) stages could deepen our understanding of these biological meanings.

## Introduction

Mild cognitive impairment (MCI) is considered a prodromal stage of Alzheimer’s disease (AD), with subjective and objective cognitive decline but preserved ability to perform independent daily activities (Petersen and Negash, 2008). In particular, amnestic MCI (aMCI) patients, whose main symptom is a decrease in memory function among cognitive function items, are reported to progress to dementia at a rate of 10–15% every year (Gauthier et al., 2006). Recently, the aMCI stage has been subdivided into early and late MCI based on the degree of delayed memory recall impairment (Jessen et al., 2014). It is estimated that early MCI reflects an earlier stage of AD progression, and late MCI is reported to have a higher risk for AD progression than early MCI (Jessen et al., 2014). Furthermore, attempts have been made to explore the biological basis of neurodegeneration in the aMCI stage, subdivided based on memory performance. In our previous study, cortical atrophy in healthy controls (HC), early-MCI patients, and late-MCI patients was compared by whole-brain analysis (Kang et al., 2019). We found that early-MCI patients show a significant atrophy in the right middle temporal gyrus compared with that in HC, and late-MCI patients showed a greater atrophy in the left fusiform gyrus than did early-MCI patients. Additionally, it has been established that the association between brain volume and memory decline in these regions is significantly different depending on MCI subdivided stage (Kang et al., 2019). With regard to resting-state brain functional connectivity (FC), a representative biomarker for AD progression (Liu et al., 2008), the mean FC value of the default-mode network (DMN) has shown a significant difference among HC, early-MCI patients, and late-MCI patients (Lee et al., 2016). However, it was difficult to identify patterns showing differences between the three groups by only confirming the regions showing differences in mean DMN FC between two of the three groups. Moreover, since the interaction between group and cognitive function was not analyzed, the differential relationship between FC and cognitive function was not evaluated as the prodromal stage progressed (Lee et al., 2016). Finally, because FC was evaluated only within the DMN, it was impossible to evaluate FC between other brain regions. A previous study that performed seed-based FC analysis using the thalamus as a seed region has indicated that FC between regions such as the bilateral superior temporal gyrus, right fusiform gyrus, and thalamus decreased in early and late MCI compared to that in HC (Cai et al., 2015). However, FC between the left FG, bilateral precuneus, and thalamus was increased. The thalamus-seeded FC difference was confirmed between early and late MCI. However, no significant association was found between FC and cognitive function (Cai et al., 2015). This study did not also evaluate whether this relationship varies with the progression of AD stage.

The posterior cingulate cortex (PCC) is a hub region of the DMN, showing the highest metabolic rates in healthy older adults compared to other brain regions (Raichle and Snyder, 2007), and is vulnerable to amyloid-beta deposition induced by an increased neuronal activity of this brain region in the early phases of AD (Zott et al., 2019). Prior research has demonstrated that intra-regional FC and inter-regional FC of PCC reflect AD progression (Liu et al., 2008). In addition, another previous study has found decreased FC of PCC with the temporal cortex but increased FC with the frontal cortex in aMCI patients compared to cognitively intact healthy older adults (Bai et al., 2009). Moreover, FC between PCC and the middle temporal gyrus shows a significant correlation with working memory and executive function in aMCI patients (Bai et al., 2009). Despite the clinical implications of PCC in prodromal AD, there has been little discussion about the functional brain changes in early- and late-aMCI stages. Furthermore, there has also been no study to examine the variation in correlation between memory performance and FC in the aMCI stage according to subdivided aMCI stage. Through an investigation of these unexplored issues, we hope to gain a deeper understanding of how changes in brain function in early- and late-MCI stages interact with cognitive decline.

In this study, we aimed to compare the FC from the PCC between cognitively healthy older adults, early-aMCI patients, and late-aMCI patients by whole-brain analysis. In addition, this paper assesses whether the association between the FC of PCC with regions of interest (ROIs) and memory performance differs according to the subdivided aMCI stage of early and late aMCI.

## Materials and Methods

### Participants

One hundred two subjects were included in this study [37 subjects in the healthy control group (age range: 72–78 years), 30 subjects with early MCI (age range: 71–82 years), and 35 subjects with late MCI (age range: 69–82 years)]. The subjects were recruited from the Catholic Geriatric Brain MRI database, which was built through the outpatient psycho-geriatric clinic of Seoul Saint Mary’s Hospital located in Seoul, South Korea, from October 2016 to July 2018. The cognitive functions of all subjects were assessed with the Korean version of the Consortium to Establish a Registry for AD (CERAD-K) (Lee et al., 2002). The measures included assessment in verbal fluency (VF), the 15-item Boston Naming Test (BNT), the Korean version of the Mini-Mental State Examination (MMSE-K) (Park, 1989), word list memory (WLM), word list recall (WLR), word list recognition (WLRc), constructional praxis (CP), and constructional recall (CR). In addition, total memory (TM) domain scores were obtained by summing the scores from the CERAD-K, WLM, WLR, and WLRc. Patients with MCI met Peterson’s criteria of (1) memory complaint corroborated by an informant, (2) objective memory impairment for age, level of education, and sex, (3) essentially preserved general cognitive function, (4) mostly intact functional activities, and (5) no dementia. All MCI patients had an overall clinical dementia rating of 0.5 (Morris, 1993). The classifications of late MCI and early MCI were as follows: subjects classified with late MCI reported memory impairment, demonstrated by memory performance scores greater than 1.5 standard deviations (SDs) below the respective age-, education-, and sex-specific normative mean on the CERAD-K WLR; on the other hand, subjects classified with early MCI had performance scores between 1.5 and 1.0 SDs below the normative mean. Concise descriptions of the tests and the review process are described in the Supplementary Material. We excluded participants with any history of alcoholism, drug abuse, head trauma, or psychiatric disorders; those with multiple vascular risk factors, extensive cerebrovascular disease, or taking any psychotropic medications (e.g., cholinesterase inhibitors, antidepressants, benzodiazepines, and antipsychotics) were excluded. The inclusion criteria for elderly HCs were as follows: (1) older than 60 years of age, (2) within 1.0 SD for the CERAD-K WLR and within 1.5 SDs on the other domains of the CERAD-K, and (3) a clinical dementia rating score of 0. The study was conducted under the ethical and safety guidelines set forth by the Institutional Review Board of The Catholic University of Korea, which approved all study procedures. Informed and written consent was obtained from all participants.

### MRI Acquisition

Imaging data were collected in the Department of Radiology of Seoul Saint Mary’s Hospital at The Catholic University of Korea using a 3T Siemens Verio machine and an eight-channel Siemens head coil (Siemens Medical Solutions, Erlangen, Germany). The parameters used for T1-weighted volumetric magnetization-prepared rapid gradient-echo scan sequences were echo time = 2.5 ms, repetition time = 1,900 ms, inversion time = 900 ms, field of view = 250 mm, matrix = 256 × 256, and voxel size = 1.0 × 1.0 × 1.0 mm^3^. The resting-state functional images were collected using a T2-weighted gradient-echo sequence with the following parameters: TR = 2,490 ms, TE = 30 ms, matrix = 128 × 128 × 29, and voxel size = 2 × 2 × 3 mm^3^. One hundred fifty volumes were acquired over 5 min with the instruction: “Keep your eyes closed and think of nothing in particular.”

### Image Preprocessing

We used the Data Processing Assistant for Resting-State fMRI (DPARSF) (Chao-Gan and Yu-Feng, 2010), which is based on Statistical Parametric Mapping (SPM^1^), to preprocess the fMRI images. Slice timing and realignment for motion corrections were performed on the images. Subjects with excessive head motion (cumulative translation or rotation > 2 mm or 2°) were excluded. To prevent group-related differences caused by microscopic head motion, we compared framewise displacement (FD) between groups. The mean FD scores did not differ between groups (*p* > 0.05, two-sample *t*-test), and they were further used as covariates for group comparisons. For spatial registration, the T1-weighted image was co-registered to the mean resting-state functional MRI based on rigid body transformation. For spatial normalization, the International Consortium for Brain Mapping template was applied (resampling voxel size = 3 × 3 × 3 mm^3^) and fitted to the “East Asian brain.” After this, functional images were spatially smoothed with a 6-mm full-width at half-maximum Gaussian kernel.

We further processed our functional data so they fit the FC analysis with the DPARSF. Linear trends were removed from the functional images, and data were filtered with a temporal band-pass of 0.01–0.08 Hz. This filtering reduces low-frequency drift as well as physiological high-frequency respiratory and cardiac noise (Biswal et al., 1995).

### Functional Connectivity Analyses

Seed-based FC analysis was used to construct resting-state networks. A spherical ROI (radius = 6 mm) was centered at the given coordinates within the PCC as a seed region [(MNI) space: 0, –51, and 29] (Greicius et al., 2003; Sestieri et al., 2011). For each subject, the mean time series of each seed region was computed as the reference time course for each network. Pearson cross-correlation analysis was performed between the seed time course and the time course of the whole-brain voxels. A Fisher’s z-transformation was applied to improve the normality of the correlation coefficients (Wang et al., 2006). Finally, the individual maps of each network were obtained.

### Statistical Analysis

Statistical analyses for demographic data were performed with R software (version 2.15.3). Normality assumptions were tested for all continuous variables using the Kolmogorov–Smirnov test. All variables were normally distributed. One-way ANOVA and chi-square (χ^2^) test were used to assess potential differences between the HC, early-MCI, and late-MCI groups for all demographic variables. All statistical analyses used a two-tailed level of 0.05 for defining statistical significance. We conducted a whole-brain voxel-wise analysis of between-group differences in FC with a general linear model using SPM8, controlling for age, sex, education years, apolipoprotein E (*APOE*) genotype, and voxel-wise gray matter (GM) volume (Oakes et al., 2007). The process of obtaining GM intensity maps in the MNI space is described in the Supplementary Material. Multiple corrections were performed using cluster-extent correction (AlphaSim) as implemented by Data Processing and Analysis for Brain Imaging, and the following parameters were set: *p* < 0.01 for statistical significance in individual voxel threshold comparison, 1,000 Monte Carlo simulations, and *p* < 0.01 as the effective threshold for cluster-extent correction. Subsequently, the identified brain regions showing significant differences in FC compared to that of the seed region or between groups were extracted as ROIs. In *post hoc* procedures, Z-transformed FC (zFC) values between seed regions and ROIs were used to compare region-specific differences in FC between groups (HC *vs*. early MCI, HC *vs*. late MCI, and early MCI *vs*. late MCI), with Bonferroni multiple testing correction.

To assess the main effect of the interaction between group and cognitive performance on zFC values between the PCC and ROIs, we used multiple regression analyses after adjusting for age, sex, education years, and *APOE* genotype. We applied a threshold of α = 0.05 to consider regression weights significant, and we additionally accounted for multiple testing using the Bonferroni correction for each hypothesis.

## Results

### Baseline Demographic and Clinical Data

Table 1 shows the baseline demographic data for the different subject groups. There were no significant differences in sex, years of education, and *APOE* ε4 genotype between the control, early-MCI, and late-MCI groups. However, there was a significant difference in age across groups (*p* < 0.001). As expected based on the inclusion criteria, the groups differed in memory performance test scores (MMSE-K, CERAD-K WLM, WLR, WLRc, and TM) (*p* < 0.001). Concerning non-amnestic cognitive functions, there were significant differences in CERAD-K VF, BNT, CR (*p* < 0.001), and CP scores across groups (*p* = 0.024) (see Supplementary Table 1).

**TABLE 1:** Demographic and clinical characteristics of the study participants.

	Control group (*n* = 37)	Early-mild cognitive impairment (MCI) group (*n* = 30)	Late MCI group (*n* = 35)	*P*-value
Age (years)	73.9 ± 2.0 (72–78)	76.9 ± 4.3 (71–82)	77.3 ± 4.1 (69–82)	<0.001
Sex (M/F,%)	40.5: 59.5	53.3: 46.7	45.7: 54.3	0.579
Education (years)	10.8 ± 4.0 (4–16)	9.0 ± 4.8 (2–20)	9.6 ± 4.3 (2–16)	0.259
*APOE* ε4 carrier (*n*,%)	8 (21.6%)	13 (43.3%)	14 (40.0%)	0.121
MMSE-K	27.1 ± 1.7 (24–30)	21.7 ± 4.3 (14–30)	21.7 ± 4.5 (11–29)	<0.001
CERAD-K WLM	17.8 ± 3.3 (12–25)	12.8 ± 4.4 (6–20)	10.6 ± 4.1 (3–19)	<0.001
CERAD-K WLR	6.0 ± 1.7 (4–9)	2.5 ± 0.8 (2–5)	0.9 ± 1.0 (0–3)	<0.001
CERAD-K WLRc	9.2 ± 0.8 (8–10)	6.6 ± 2.2 (3–10)	5.2 ± 2.6 (0–10)	<0.001
CERAD-K TM	33.0 ± 5.0 (25–47)	21.9 ± 6.0 (12–32)	16.7 ± 5.7 (6–29)	<0.001

*The data are presented as mean ± SD (minimum–maximum) unless indicated otherwise. MMSE-K, Korean version of the Mini-Mental Status Examination; CERAD-K, Korean version of the Consortium to Establish a Registry for Alzheimer’s disease; WLM, word list memory; WLR, word list recall; WLRc, word list recognition; TM, total scores of memory domains, including CERAD-K WLM, WLR, and WLRc.*

### Group Differences in Functional Connectivity: Whole-Brain Voxel-Based Analysis

**FIGURE 1:**
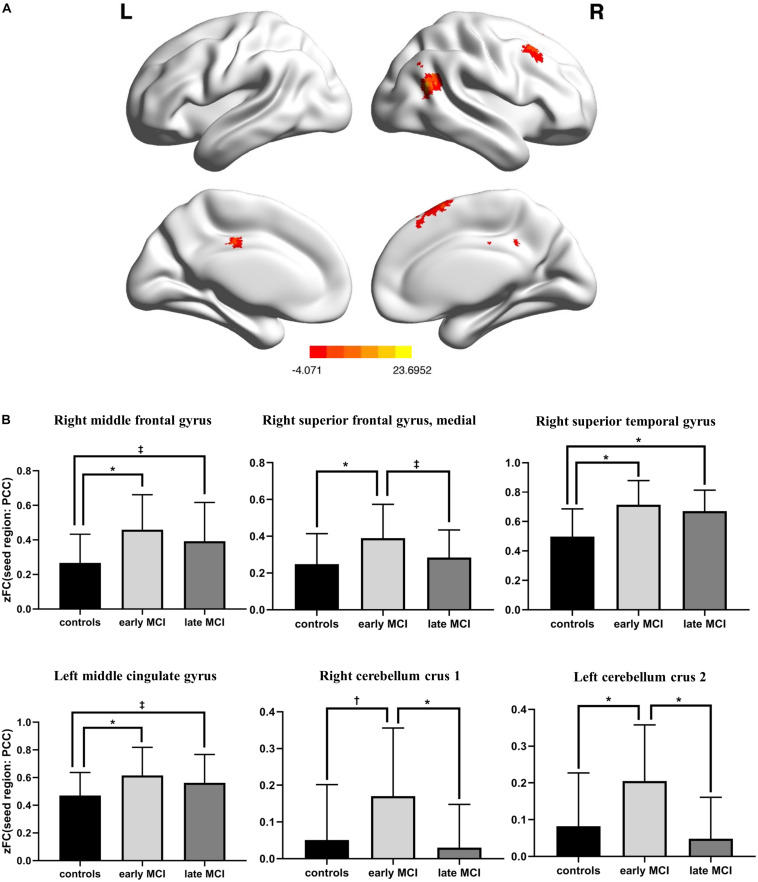
**(A)** Brain regions where the functional connectivity of the posterior cingulate cortex showed significant differences among control subjects, early-mild cognitive impairment (MCI) patients, and late-MCI patients (ANCOVA, Monte Carlo simulation-corrected *p* < 0.01). **(B)** Difference in functional connectivity from the posterior cingulate cortex among control subjects, early-MCI patients, and late-MCI patients (Bonferroni *post hoc* tests). ^∗^Bonferroni-corrected *P* < 0.001; †Bonferroni-corrected *P* < 0.01; ‡Bonferroni-corrected *P* < 0.05.

There was a significant difference in the zFC of PCC with right middle frontal gyrus, superior medial frontal gyrus, superior temporal gyrus, left middle cingulate gyrus, right cerebellum crus 1, and left cerebellum crus 2 among HC, early-MCI patients, and late-MCI patients (Figure 1A and Table 2). Additionally, in the right middle frontal gyrus, superior temporal gyrus, and left middle cingulate gyrus, early- and late-MCI groups showed significantly higher zFC values than the HC (Bonferroni-corrected *p* < 0.05), but there was no significant difference between early- and late-MCI patients (Figure 1B). In the right superior medial frontal gyrus, cerebellum crus 1, and left cerebellum crus 2, the early-MCI patients displayed higher zFC values than the HC and late-MCI patients (Bonferroni-corrected *p* < 0.05, Figure 1B).

**TABLE 2:** Anatomical locations of regions showing significant differences in functional connectivity from the posterior cingulate cortex among control subjects, early-mild cognitive impairment (MCI) patients, and late-MCI patients.

Region	L/R	Cluster (voxel count)	Peak *F* value	Peak MNI coordinates (*x*, *y*, *z*)		
Middle frontal gyrus	R	60	11.0123	39	18	42
Superior frontal gyrus, medial	R	53	10.2437	9	30	51
Superior temporal gyrus	R	189	23.6952	45	−57	21
Middle cingulate gyrus	L	95	10.4025	−3	−21	36
Cerebellum crus 1	R	83	9.8205	21	−66	−33
Cerebellum crus 2	L	300	11.2579	0	−81	−33

*FC, functional connectivity; PCC, posterior cingulate cortex; L, left; R, right.*

### Interaction of Memory Performances and Functional Connectivity Between PCC and ROIs

**FIGURE 2:**
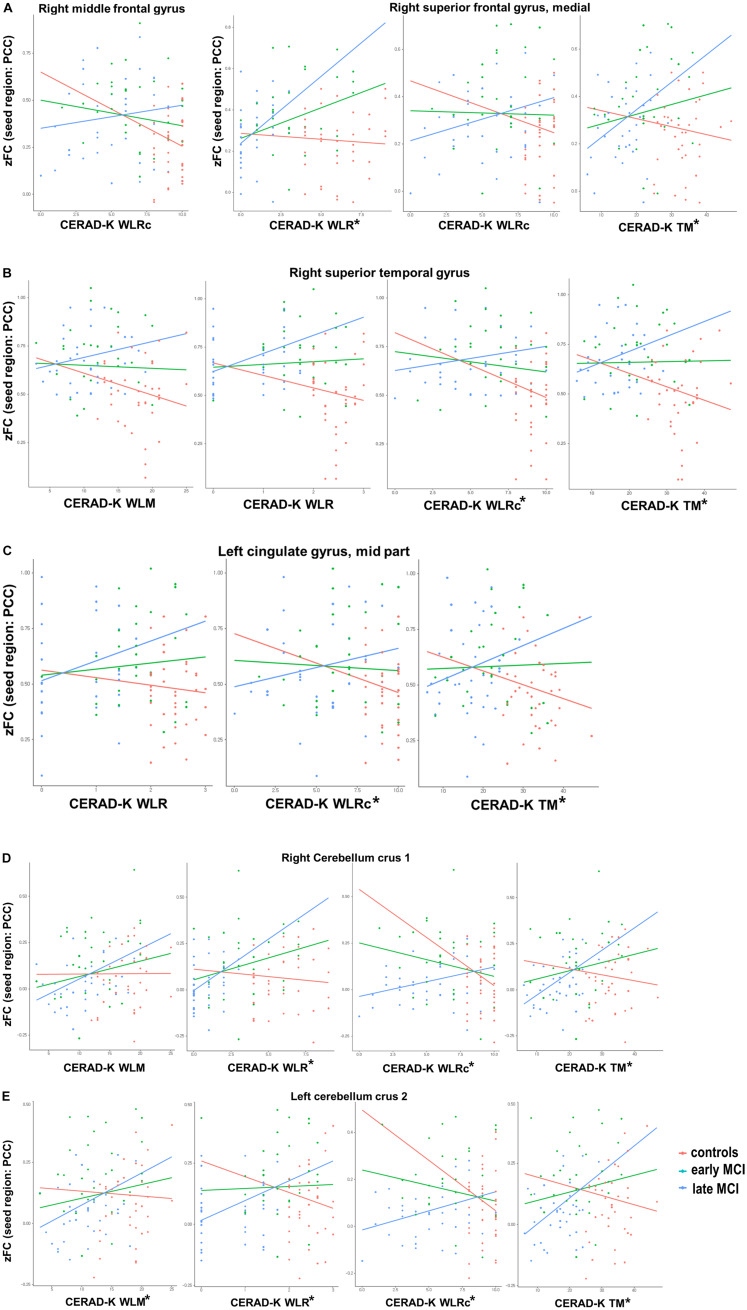
Significant group [healthy control *vs*. early-mild cognitive impairment (MCI) *vs*. late MCI] by cognitive performance interactions for functional connectivity between posterior cingulate cortex and **(A)** right frontal lobe, **(B)** right superior temporal gyrus, **(C)** left cingulate gyrus—mid part, and **(D,E)** bilateral cerebellum. CERAD-K, Korean version of the Consortium to Establish a Registry for Alzheimer’s disease; VF, verbal fluency; BNT, Boston Naming Test; WLM, word list memory; WLR, word list recall; CR, constructional recall; TM, total scores of memory domains, including CERAD-K WLM, WLR, and WLRc; *Bonferroni-corrected *P* < 0.05 by multiple regression analysis.

With regard to the CERAD-K WLM, we found a significant group interaction for zFC scores of PCC with the right superior temporal gyrus, right cerebellum crus 1, and left cerebellum crus 2. When we applied the Bonferroni correction, the results remained significant only for the zFC values of PCC with the left cerebellum crus 2. Concerning the CERAD-K WLR, there was a significant group interaction for the zFC scores of PCC with the right superior medial frontal gyrus, superior temporal gyrus, left middle cingulate gyrus, right cerebellum crus 1, and left cerebellum crus 2. With Bonferroni multiple testing correction, the results remained significant for the zFC values with the right superior medial frontal gyrus, right cerebellum crus 1, and left cerebellum crus 2. In addition, there was a significant group interaction between the CERAD-K WLRc score and the zFC of PCC with the right middle frontal gyrus, superior medial frontal gyrus, superior temporal gyrus, left middle cingulate gyrus, right cerebellum crus 1, and left cerebellum crus 2. However, after performing Bonferroni correction, a significant interaction remained only for zFC values with the right superior temporal gyrus, left middle cingulate gyrus, right cerebellum crus 1, and left cerebellum crus 2. Lastly, there was a significant group interaction between the CERAD-K TM score and the zFC with the right superior medial frontal gyrus, superior temporal gyrus, left middle cingulate gyrus, right cerebellum crus 1, and left cerebellum crus 2 after Bonferroni correction. Figure 2 provides an overview of significant group-memory performance interactions on FC between PCC and ROIs (HC *vs*. early MCI *vs*. late MCI).

## Discussion

The current study sets out to evaluate brain regions showing a significant difference in FC from the PCC, the representative hub region of the DMN, between cognitively intact older adults and early- and late-aMCI patients by whole-brain analysis. In addition, this research explored whether the relationships between the FC of PCC with ROIs and memory performance differ according to the progression of prodromal AD.

The early-MCI patients displayed a higher zFC from the PCC in the right superior medial frontal gyrus and bilateral cerebellum crus than did the HC and late-MCI patients. Additionally, the highest zFC was found from the PCC with the right middle frontal gyrus, superior temporal gyrus, and left middle cingulate gyrus in the early-MCI group, while the difference between early- and late-MCI groups was not significant. In a previous study that analyzed the mean FC of DMN through independent component analysis (ICA), contrary to the results of the present study, the early-MCI patients showed a lower FC of DMN than did HC (Lee et al., 2016). However, in agreement with the present study, the late-MCI patients showed a lower FC than that of early-MCI patients (Lee et al., 2016). However, the previous study had extracted only the FC values within the DMN brain regions through the ICA and not FC with other brain regions (Lee et al., 2016). These results, therefore, need to be interpreted with caution. Additionally, another previous study presented different results in longitudinal change in FC among subdivided MCI stages (Zhan et al., 2016). In this prior paper, the degree of change in the nodal FC of the right putamen was smallest in late MCI, and that in the nodal FC of the right insula was smallest in early MCI (Zhan et al., 2016). Moreover, the inter-network FC between the DMN and salience network decreased sequentially as CN progressed to late MCI (Zhan et al., 2016). However, considering that the average disease duration for MCI stage is roughly 4 years at the average age of the participants in the previous study (mean age of 70) (Vermunt et al., 2019), the follow-up period of 6 months is not sufficient, and the sample size is small. Therefore, it is important to consider the possible bias for interpreting these results.

Concerning the ROIs, the right superior medial frontal gyrus of MCI patients has been demonstrated to have a higher FC with the right middle fusiform gyrus during face matching tasks compared to that of the HC group (Bokde et al., 2006). Additionally, increased intra-regional brain activity in this brain region has been suggested to perform a compensatory role in the progression of AD (Yuan et al., 2016). Furthermore, previous results on increased activity in this ROI with higher levels of education might support this compensatory function (Boller et al., 2017). Given the impacts of Aβ retention and *APOE* ε4 allele genotype on the increased functional activity of this ROI (Oh et al., 2014; Wang et al., 2015), further studies with these AD risk factors should be undertaken.

The cerebellum crus, another ROI, belongs to lobule VII of the posterior lobe of the cerebellum (D’Mello and Stoodley, 2015), where abundant Aβ retention and increased microglia are observed in AD patients (Sjöbeck and Englund, 2001; Wang et al., 2002). In the present study, FC between the PCC and bilateral cerebellum crus showed inverse-U-shaped change patterns in all groups. Previous research has found increased FC of the posterior cerebellum with the temporal and parietal cortex at baseline but greater decreased FC between the posterior cerebellum and the aforementioned ROIs after 20 months (Bai et al., 2011). That change in FC over time might have contributed to the inverse U-shaped curve found in the current study. Additionally, previous research has suggested that the posterior cerebellum might have a compensatory role against functional impairment of the hippocampus, given the negative association between changes in the intra-regional activity of these two brain regions (Bai et al., 2011).

The present study found a differential relationship between memory performance and FC from PCC with ROIs according to MCI stage. Although the present study is cross-sectional and cognitive decline does not necessarily indicate progression of AD with associated decreased memory performance, the HC group showed increased FC, while the MCI group showed decreased FC in most of the ROIs. In addition, the linear regression coefficient between FC and memory performance was higher in the late-MCI group. These results indicate that the compensatory role of FC weakens as HC progress to late MCI. Furthermore, the highest FC of early-MCI patients in various ROIs could be induced by other factors, including Aβ accumulation (Zott et al., 2019), rather than by a compensatory mechanism (Wang et al., 2006; Yuan et al., 2016). In this regard, further studies considering these variables are needed. These results are of clinical significance in that there have been few previous studies examining the interaction between memory performance and FC in subdivided MCI stages. In a previous paper, although participants in HC and early- and late-MCI groups have exhibited a positive association between the mean FC of DMN and cognitive function, such as memory performance and executive function, it was uncertain whether this association varies by group (Lee et al., 2016).

Among the ROIs that showed a significant group-memory performance interaction for FC from PCC, a previous study has shown that the brain activity of the medial superior frontal gyrus is increased by memory encoding strategy training, supporting the compensatory function of this brain region (Kirchhoff et al., 2012). Concerning the superior temporal gyrus, the FC with the hippocampus was disrupted in mild AD compared to the normal group (Wang et al., 2006). In the current results, a decrease in FC with cognitive decline was not noticeable in early-MCI patients but significantly decreased in late-MCI patients, showing a pattern similar to that of AD. In addition, the middle cingulate cortex, another significant ROI, has been reported to be a critical hub region of the salience (SAL) network (Xie et al., 2012). Therefore, the FC between the PCC and the middle cingulate cortex observed in this study could reflect the internetwork connectivity between the DMN and the SAL network. Furthermore, the previous study has shown that the change in DMN–SAL inter-network connectivity is disrupted with progression from CN to the late MCI stage, and a disrupted inter-network connectivity shows a significant relationship with cognitive dysfunction (Zhan et al., 2016). However, since this previous study did not examine the group-by-FC-change interaction for cognitive decline, additional studies are needed to confirm whether significant differences exist in this relationship depending on subdivided MCI stage. In another prior study that analyzed the difference in FC of DMN between HC and aMCI groups, the activity of the middle cingulate cortex was increased in the aMCI group, but there was no significant association with cognitive function (Jin et al., 2012). However, considering the small sample size of the previous research, caution must be applied when interpreting the results. FC between the posterior cerebellum and DMN has been associated with cognitive function in AD patients (Zheng et al., 2017), similar to the positive association found in late-MCI patients in the present study. However, few studies have explored the differential association of FC with memory performance at the subdivided aMCI stage. Given the results of the present study, it might be assumed that the activated compensatory mechanism in the HC group weakens as aMCI progresses.

This study was limited by the absence of analysis of AD pathology, including Aβ and tau deposits, which have been demonstrated to affect cognitive decline and functional brain changes throughout the trajectory of AD (Canuet et al., 2015; Sperling et al., 2019). Therefore, further investigation and experimentation with these AD pathologies are recommended to understand the role of functional brain change in the subdivided stages of prodromal AD.

## Conclusion

The purpose of the current study was to determine the difference in FC from PCC, the hub region of the DMN, between HC and early- and late-MCI patients and to assess the differential association of FC with memory performance according to the subdivided stage of aMCI. This study illustrated a significant difference in FC from PCC to several brain regions, which is known to have a compensatory role against cognitive decline and confirmed the significant memory performance-by-group interaction for FC between PCC and ROIs. These findings contribute to the biological implications of the early- and late-aMCI stages, which are categorized by evaluating the impairment of memory performance. Additionally, complementing the aforementioned limitations and comprehensively analyzing the results of structural differences in subdivided aMCI stages could deepen our understanding of these biological meanings.

## Data Availability Statement

The datasets generated or analyzed during the current study are not publicly available due to Patient Data Management Protocol of Seoul Saint Mary’s Hospital but are available from the corresponding author on reasonable request.

## Ethics Statement

The studies involving human participants were reviewed and approved by the Institutional Review Board of The Catholic University of Korea. The patients/participants provided their written informed consent to participate in this study.

## Author Contributions

DK contributed to the conceptualization, methodology, data curation, writing—original draft, visualization, formal analysis, and funding acquisition. S-MW and YU contributed to the methodology, data curation, and writing—review and editing. H-RN contributed to the investigation and visualization. N-YK contributed to the methodology and data curation. CL contributed to the conceptualization and supervision. HL contributed to the conceptualization, methodology, writing—review and editing, supervision, project administration, and funding acquisition. All authors contributed to the article and approved the submitted version.

## Conflict of Interest

The authors declare that the research was conducted in the absence of any commercial or financial relationships that could be construed as a potential conflict of interest.

## Footnotes

1 http://www.fil.ion.ucl.ac.uk/spm
