# Supplementary material for: Distinctive Association of the Functional Connectivity of the Posterior Cingulate Cortex on Memory Performances in Early and Late Amnestic Mild Cognitive Impairment Patients
Source: Front Aging Neurosci. 2021 Jul 1;13:696735. doi: 10.3389/fnagi.2021.696735 (PMC8281268; doi:10.3389/fnagi.2021.696735)
Supplement: Supplementary file 1 [file Data_Sheet_1.docx]

Supplementary Material

**Distinctive association of the functional connectivity of the posterior cingulate cortex on memory performances in early and late amnestic mild cognitive impairment patients**

Dong Woo Kang^a^, Sheng-Min Wang^b^, Yoo Hyun Um^a^, Hae-Ran Na^b^, Nak-Young Kim^d^, Chang Uk Lee^a^, and Hyun Kook Lim^b*^

^a^Department of Psychiatry, Seoul St. Mary’s Hospital, College of Medicine, The Catholic University of Korea, Seoul, Republic of Korea

^b^Department of Psychiatry, Yeouido St. Mary’s Hospital, College of Medicine, The Catholic University of Korea, Seoul, Republic of Korea

^c^Department of Psychiatry, St. Vincent’s Hospital, College of Medicine, The Catholic University of Korea, Seoul, Republic of Korea

^d^Department of Psychiatry, Keyo Hospital, Uiwang, Republic of Korea

***Correspondence:** Hyun Kook Lim, MD, PhD

Department of Psychiatry, Yeouido St. Mary’s Hospital, College of Medicine, The Catholic University of Korea, 10, 63-ro, Yeongdeungpo-gu, Seoul, 07345, Republic of Korea

Tel: +82-2-3779-1048, Fax: +82-2-780-6577, E-mail: [drblues@catholic.ac.kr](mailto:drblues@catholic.ac.kr)

# Supplementary Methods

## Neuropsychological evaluation

# Cognitive status was assessed by neuropsychological testing at Seoul St. Mary’s Hospital, The Catholic University of Korea. The cognitive functions of all the subjects were assessed with the Korean version of the Consortium to Establish a Registry for Alzheimer’s Disease (CERAD-K), which included Verbal Fluency (VF), the 15-item Boston Naming Test (BNT), MMSE-K, Word List Memory (WLM), Word List Recall (WLR), Word List Recognition (WLRc), Constructional Praxis (CP), and Constructional Recall (CR). The CERAD is the standardized clinical and neuropsychological assessment battery for the evaluation of patients with Alzheimer's disease. The results were reviewed by a neuropsychologist to determine whether there was evidence of cognitive impairment.

# The VF score is the number of animal names that the subject could name in one minute. The BNT score ranges from 0 to 15 points. The MMSE-K score ranges from 0 to 30 points. The WLM score ranges from 0 to 30 points. The WLR score ranges from 0 to 10 points. The WLR score ranges from 0 to 10 points. The WLRc score ranges from 0 to 10 points. The CP score ranges from 0 to 11 points. The CR score ranges from 0 to 11 points.

## *APOE* genotyping

DNA was isolated from blood using the QIAmp Blood DNA Maxi Kit protocol (Qiagen, Valencia, CA). Genotypes for two APOE SNPs, rs429358 (E*4) and rs7412 (E*2) were determined using TaqMan SNP genotyping assays (Applied Biosystems, Foster City, California).

## Process of obtaining GM intensity maps

We processed the data using the VBM8 toolbox (http://dbm.neuro.uni-jena.de/vbm8/), which was part of the SPM8 software package (https://www.fil.ion.ucl.ac.uk/spm/software/spm8), Wellcome Department of Imaging Neuroscience, London, United Kingdom. Data pre-processing and analysis were performed using SPM8. Data pre-processing involved visual inspection of the T1-weighted images to control for imaging artifacts and the consecutive segmentation into gray matter (GM), white matter (WM), and cerebrospinal fluid (CSF), building a customized template for GM and WM through an iteratively non-linear registration algorithm (DARTEL Toolbox for SPM8) and a normalization of this template to the Montreal Neurological Institute template. The Jacobian determinants resulting from the normalization procedure were used to obtain modulated VBM data preserving regional volumes. Individual GM and WM images were smoothed with an isotropic Gaussian kernel of 6 mm full-width at half-maximum prior to statistical analyses.

# Supplementary Tables

|  | Control group (n=37) | Early MCI group (n=30) | Late MCI group (n=35) | *P* value |
| --- | --- | --- | --- | --- |
| CERAD-K VF | 15.0 ± 3.5 (10-23) | 10.4 ± 3.7 (6-20) | 10.2 ± 3.1 (6-17) | < 0.001 |
| CERAD-K BNT | 12.6 ± 1.7 (9-15) | 9.2 ± 3.0 (4-15) | 8.7 ± 2.8 (2-14) | < 0.001 |
| CERAD-K CP | 10.5 ± 0.7 (9-11) | 9.2 ± 1.7 (5-11) | 9.7 ± 1.3 (7-11) | 0.024 |
| CERAD-K CR | 7.2 ± 2.3 (3-11) | 2.8 ± 3.0 (0-10) | 2.1 ± 1.9 (0-6) | < 0.001 |

**Supplementary Table 1.** Non-amnestic cognitive function of the study participants. Data are presented as the means ± SD (minimum-maximum) unless indicated otherwise. CERAD-K, Korean version of Consortium to Establish a Registry for Alzheimer’s Disease; VF, Verbal Fluency; BNT, 15-item Boston Naming Test; CP, Constructional praxis; CR, Constructional Recall.
